# High Resolution Models of Transcription Factor-DNA Affinities Improve *In Vitro* and *In Vivo* Binding Predictions

**DOI:** 10.1371/journal.pcbi.1000916

**Published:** 2010-09-09

**Authors:** Phaedra Agius, Aaron Arvey, William Chang, William Stafford Noble, Christina Leslie

**Affiliations:** 1 Computational Biology Program, Memorial Sloan-Kettering Cancer Center, New York, New York, United States of America; 2 Department of Genome Sciences, University of Washington, Seattle, Washington, United States of America; Duke University, United States of America

## Abstract

Accurately modeling the DNA sequence preferences of transcription factors (TFs), and using these models to predict *in vivo* genomic binding sites for TFs, are key pieces in deciphering the regulatory code. These efforts have been frustrated by the limited availability and accuracy of TF binding site motifs, usually represented as position-specific scoring matrices (PSSMs), which may match large numbers of sites and produce an unreliable list of target genes. Recently, protein binding microarray (PBM) experiments have emerged as a new source of high resolution data on *in vitro* TF binding specificities. PBM data has been analyzed either by estimating PSSMs or via rank statistics on probe intensities, so that individual sequence patterns are assigned enrichment scores (E-scores). This representation is informative but unwieldy because every TF is assigned a list of thousands of scored sequence patterns. Meanwhile, high-resolution *in vivo* TF occupancy data from ChIP-seq experiments is also increasingly available. We have developed a flexible discriminative framework for learning TF binding preferences from high resolution *in vitro* and *in vivo* data. We first trained support vector regression (SVR) models on PBM data to learn the mapping from probe sequences to binding intensities. We used a novel 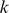-mer based string kernel called the di-mismatch kernel to represent probe sequence similarities. The SVR models are more compact than E-scores, more expressive than PSSMs, and can be readily used to scan genomics regions to predict *in vivo* occupancy. Using a large data set of yeast and mouse TFs, we found that our SVR models can better predict probe intensity than the E-score method or PBM-derived PSSMs. Moreover, by using SVRs to score yeast, mouse, and human genomic regions, we were better able to predict genomic occupancy as measured by ChIP-chip and ChIP-seq experiments. Finally, we found that by training kernel-based models directly on ChIP-seq data, we greatly improved *in vivo* occupancy prediction, and by comparing a TF's *in vitro* and *in vivo* models, we could identify cofactors and disambiguate direct and indirect binding.

## Author Summary

Transcription factors (TFs) are proteins that bind sites in the non-coding DNA and regulate the expression of targeted genes. Being able to predict the genome-wide binding locations of TFs is an important step in deciphering gene regulatory networks. Historically, there was very limited experimental data on the DNA-binding preferences of most TFs. Computational biologists used known sites to estimate simple binding site motifs, called position-specific scoring matrices, and scan the genome for additional potential binding locations, but this approach often led to many false positive predictions. Here we introduce a machine learning approach to leverage new high resolution data on the binding preferences of TFs, namely, protein binding microarray (PBM) experiments which measure the *in vitro* binding affinities of TFs with respect to an array of double-stranded DNA probes, and chromatin immunoprecipitation experiments followed by next generation sequencing (ChIP-seq) which measure *in vivo* genome-wide binding of TFs in a given cell type. We show that by training statistical models on high resolution PBM and ChIP-seq data, we can more accurately represent the subtle DNA binding preferences of TFs and predict their genome-wide binding locations. These results will enable advances in the computational analysis of transcriptional regulation in mammalian genomes.

## Introduction

Gene regulatory programs are orchestrated by transcription factors (TFs), proteins that coordinate expression of target genes both through direct interaction with DNA and with non-DNA-binding accessory proteins (cofactors). A recent catalog of human and mouse TFs documented almost 900 likely TFs in the human genome, including over 500 with sequence-specific binding to double-stranded DNA [1]. Accurately modeling the DNA sequence preferences of these TFs, and using these sequence preferences in an appropriate way to predict whether the TF can bind a genomic site *in vivo*, are key pieces in unraveling the regulatory code. For many years, these efforts have been frustrated by the limited availability and quality of TF binding site motifs, usually represented as a position-specific scoring matrix (PSSM) or a consensus sequence. These motifs may match thousands of sites in intergenic regions, producing an unreliable list of potential TF target genes. [2] showed that motif hits in yeast could be filtered by TF occupancy profiles measured by ChIP-chip experiments, producing a better quality regulatory map. However, TF occupancy is condition-specific and, in metazoan genomes, cell type-dependent, due to differences in chromatin state, concentrations of cofactors, and other epigenetic determinants. Since it is not feasible to collect occupancy data for all TFs and all possible cellular contexts, we must develop better methods for predicting *in vivo* occupancy, which will depend in part on improving our models of TF binding preferences.

Recently, protein binding microarray technology (PBM) has emerged as a new high-throughput technique to obtain more comprehensive data on a TF's *in vitro* sequence specificities [3]. PBM experiments measure binding of a fluorescently tagged TF or TF binding domain to a carefully designed set of double-stranded DNA probes which cover the space of all possible DNA 10-mers.

So far, PBM data has been analyzed by extracting PSSMs or computing rank statistics on probe intensities from the TF binding experiment [3]. Traditional PSSMs may underfit PBM data by failing to capture subtle but detectable sequence preferences. Alternatively, an enrichment score (E-score) can be computed for each short sequence pattern, e.g. all possible 8-mers [3] or longer gapped 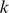-mer patterns. The collection of 8-mers with high E-scores then constitutes a kind of binding profile, with the E-score value giving a ranking of binding preferences. This representation provides much more information about a TF's DNA sequence affinities than a PSSM, but it is quite unwieldy, as each TF is assigned a list of thousands of scored 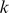-mer sequence patterns. Moreover, the E-score approach only implements a rough summarization of the raw probe-level intensity data and, in particular, treats each 8-mer (or longer gapped) pattern independently without attempting to exploit sequence similarities between 8-mers.

**Figure 1:**
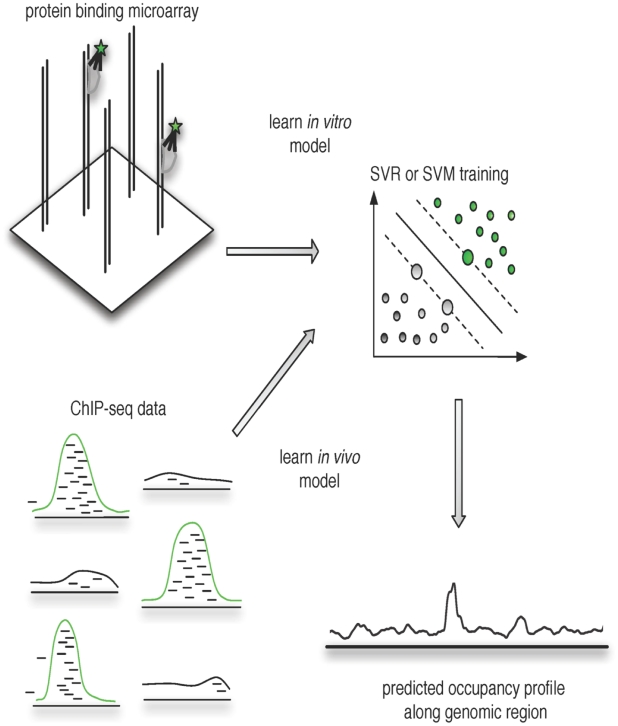
Supervised learning of TF sequence specificities from protein binding microarrays. In our approach, we directly learn the mapping from double-stranded DNA probe sequence to intensity in the PBM TF binding experiment by using support vector regression (SVR) together with novel 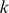-mer based string kernels. Probe sequences containing high affinity binding sites have high intensity in the PBM binding experiment; such probes are shown bound by the fluorescently tagged TF (left) and are indicated by green points in the SVR training (right). The SVR predicts probe intensity from probe sequence composition. The trained SVRs can be used to scan intergenic regions to predict *in vivo* TF occupancy.

In recent years, there have been numerous successful applications of discriminative machine learning techniques to sequence modeling problems in computational biology (reviewed in [4]), including 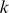-mer based string kernel methods that exploit approximate matches of short sequence patterns [5], [6]. These studies suggest that a more compact and accurate model of TF binding affinities could be *learned* from PBM data by *training* on probe sequences with a suitable kernel approach. In the first part of our study, we used a supervised learning strategy to obtain more accurate TF binding preference models from *in vitro* PBM probe-level data. As a component of this strategy, we developed a novel string kernel for comparing short double-stranded DNA sequences in a manner that captures similarity of potential TF binding sites. This kernel, called the *di-mismatch kernel*, is a first order Markov mismatch kernel – meaning that it is based on the alphabet of dinucleotides (see Materials and Methods) – and extends the 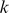-mer based string kernel methods that we and others have used for a wide range of problems involving modeling of biological sequences. In our approach, we used 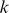-mer based string kernels for representing the similarity of double-stranded probe sequences on the PBM, and we trained support vector regression (SVR) models to directly learn the mapping from probe sequence to binding intensity from PBM training data (Figure 1, top). The trained models can then be used directly to scan intergenic regions, yielding a predicted occupancy profile (Figure 1, bottom).

To benchmark our approach, we used a large data set of mouse and yeast TFs from three separate studies for which PBM data for two independent probe designs is available. In these cases, we can train SVR models and compute E-scores using data from one PBM probe design and test how well each method predicts the high-intensity probes in the second probe design. We found that our SVR method strongly and consistently outperformed both the E-score and PSSM methods for this *in vitro* binding prediction task. Moreover, by using SVRs to score yeast intergenic regions as well as mouse and human genomic regions, we were better able to predict genomic occupancy as measured by ChIP-chip and ChIP-seq, compared with a previously described occupancy scoring method based on E-scores or PSSM-based prediction.

In the second part of our study, we trained kernel-based SVM models directly on ChIP-seq data, learning to discriminate between ChIP-seq peak and non-peak genomic regions. We call these *in vivo* models, although the ChIP-seq experiments are performed in cell lines, to distinguish them from PBM-trained *in vitro* models. We found that the ChIP-derived SVM models significantly improve TF occupancy prediction in mammalian genomes when compared to PBM-derived SVR models. Moreover, our SVM approach outperforms existing PSSM approaches such as Weeder and MDscan [7], [8]. Finally, we performed a feature analysis to extract 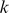-mers contained in both the *in vitro* and *in vivo* models. In the latter case, we were able to identify binding information about cofactors and disambiguate direct and indirect binding. These results suggest a strategy for combining discriminatively trained models from *in vitro* and *in vivo* data in order to decipher the transcriptional regulatory code.

## Results

### SVRs with di-mismatch kernel methods learn *in vitro* TF sequence preferences

A PBM experiment provides high resolution data on the binding affinities of a TF, comprising 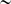
44K double stranded DNA probes and corresponding measured probe intensities, which quantify the TF binding affinities for the probe sequences. The unique sequence in each probe is a 36-mer, and the probe set is mathematically specified to contain all possible 10-mers as subsequences. We used the probe data as labeled training examples, i.e. pairs 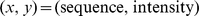, for learning a function 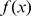 that predicts binding intensity from (36-mer) sequences. Since we were not only interested in learning to distinguish between bound and unbound probes but also predicting the range of binding affinities, we used support vector regression (SVR) to train our models.

To compare pairs of probe sequences for SVR training, we developed a novel string kernel called the di-mismatch kernel, which is a 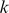-mer based string kernel adapted to the problem of TF binding models (see Materials and Methods). Briefly, this kernel computes a similarity between probe sequences based on inexact matches to 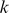-mer features, allowing up to 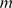 mismatches, where we count mismatches in the alphabet of dinucleotides. This choice reduces the size of the “mismatch neighborhood” of a given 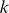-mer (i.e. fewer 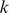-mers are similar to it) and favors mismatches that occur consecutively. A typical parameter choice is 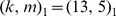, i.e., considering 13-mer sequences, allowing up to 5 mismatches, and operating in the first order alphabet of dinucleotides.

### Trained SVRs yield more accurate *in vitro* TF binding models

We first tested the performance of our SVR models on *in vitro* binding preferences, in order to establish that they could better capture TF sequence specificities than existing approaches. For 33 yeast and 114 mouse TFs, experimental data for two independent PBM array designs were available [9], [10], measuring TF binding against two completely disjoint PBM probe sets. This combined data set provided a perfect cross-validation setting where we trained a model using data from one array design (“training PBM”) and then tested the model's ability to predict binding preferences on the other array design's probe sequences (“test PBM”). We benchmarked the SVR models against the E-score approach [3], using the E-scores for all 8-mer patterns, both contiguous and gappy, as computed and posted on the Uniprobe database [11]. E-scores are modified Wilcoxon rank statistics that assess the enrichment of a given 8-mer sequence pattern in probe sequences at the top of the intensity ranking in a PBM experiment. These scores range from −0.5 to 0.5, where scores approaching 0.5 indicate that the 8-mer pattern is mostly present in bound probe sequences. In their yeast *in vivo* predictions, [9] identify high scoring 8-mer patterns to predict TF binding preferences. Therefore, we compared SVR performance to a maximum E-score approach, where each probe sequence in the test PBM is assigned the maximal E-score over the 8-mer patterns it contains, and the E-scores are computed on the training PBM. We call this the E-max score. We note that due to feature selection, our models contain no more than 4,000 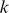-mer features. By contrast, for the data set 114 mouse TFs, the average number of 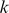-mers with E-scores above 0.35 (the threshold used for reporting the pattern) was 13,300, with just 10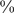 of the TFs having fewer than 3,000 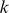-mers and 64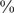 having more than 10,000 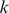-mers. In this sense, the SVR models are more compact than the E-score approach.

Because we want the models to predict the most preferred binding sequences, we first validated the results by counting how many of the top 100 predicted probes are in the top 100 highest intensity probes in the test data. Naturally, these scores range from 0 to 100, with 100 indicating perfect detection of the preferred test probes by the top predictions; we refer to these validation scores as “the detection of the top 100 probes”. For each method and each TF, we averaged the detection rates over the two PBM designs to get one representative score for the TF.

**Figure 2:**
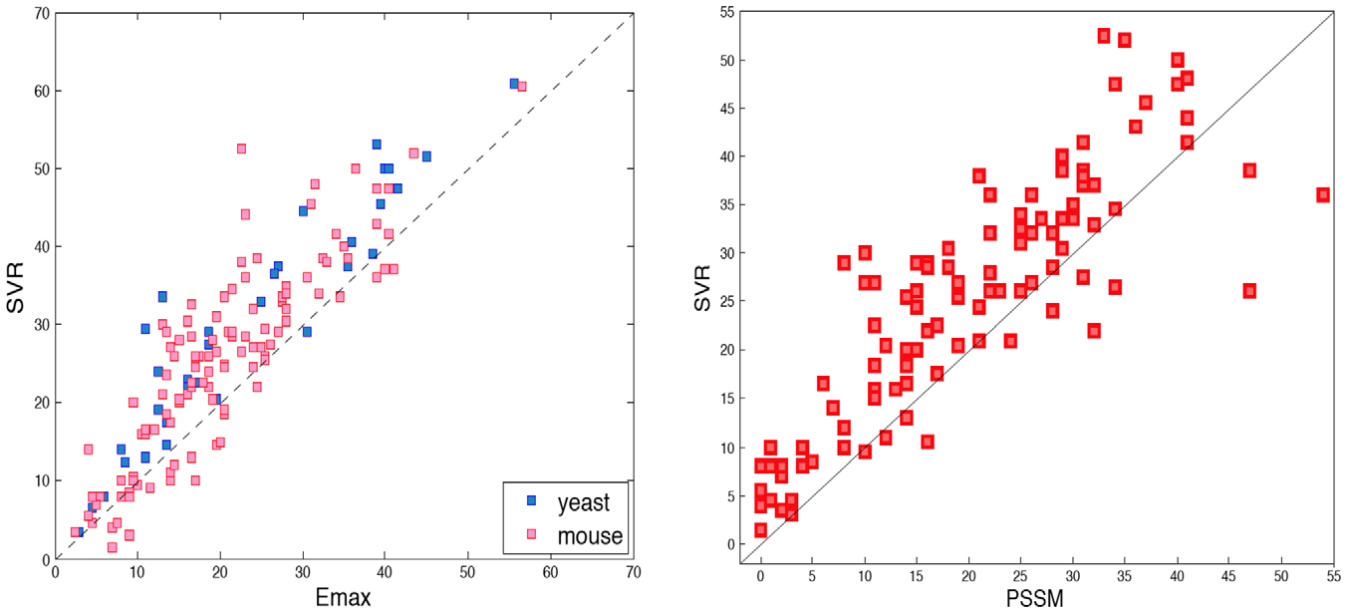
SVR models improve over E-scores and PSSMs for *in vitro* binding prediction. (a) The scatter plot shows the detection of the top 100 probes using maximum E-scores (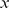-axis) and the SVR model (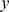-axis) in the prediction of *in vitro* TF binding preferences. Each point corresponds to one TF. The figure contains 37 yeast TFs from [12], 33 yeast TFs from [9] (blue), and 114 mouse TFs from [10] (red). (b) This panel is similar to panel (a), but compares the SVR versus PBM-derived PSSMs for the 114 mouse TFs.

To ensure that our model was not specifically tuned to the PBM array data published in the Bulyk lab, we tested our model on another set of yeast PBM arrays published by [12]. Although the PBM array design is intrinsically the same, the probe sequences are different. Two array designs were used to run experiments on 37 yeast TFs and, as before, we perform a cross-validation experiment and compare to the E-max performance by using the published E-scores for this data set. In Figure 2 (a), we show a scatter plot for the three datasets, contrasting SVR to the E-max scores for yeast and mouse PBM data. When a point lies above the diagonal line, the SVR model is better at detecting the top 100 than the E-max approach; we observe that over 80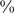 of the points lie above the diagonal (149 out of 184 TFs). This performance advantage is not achieved with standard string kernels as the spectrum and regular mismatch kernels. When we tested the mismatch kernel with parameters that intuitively seemed suitable – 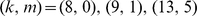 – we found little improvement over E-max and much weaker performance than the di-mismatch kernel (see Figure S4 in Text S1).

To compare our discriminative model against a standard PSSM motif approach, we also tested the performance of PBM-derived PSSMs for the mouse TF data set [10]. PSSMs for these TFs, derived from PBM probe intensity data using the Seed-and-Wobble algorithm [3], are available through the Uniprobe database. However, for the Uniprobe motifs, data from both PBM array designs for a TF have been combined to estimate a single PSSM. For a fair comparison in our cross-validation setting, we therefore re-ran the Seed-and-Wobble algorithm on each PBM experiment separately, using parameters similar to those adopted for the published motifs: we used patterns of 8-mers (allowing two gaps) in Seed-and-Wobble and then “trimmed” the resulting PSSM to maximize similarity (as measured by KL divergence) to the published PSSM. Then we used the PSSM derived from the first array design to test on probe sequences from the second array design, and vice versa. We found that SVR strongly outperforms PSSMs (Figure 2 (b) with wins on 81% of the TFs, while E-max essentially ties the PSSM performance (E-max wins on 52% of TFs, Figure S1 in Text S1), suggesting that E-max and PSSM approaches are similar and correlated. We note that since the Seed-and-Wobble method uses on E-scores to derive PSSMs, this correlation is perhaps expected.

We note that other algorithms for extracting PSSMs from PBMs have also been proposed, including RankMotif++ [13] (see Figure S3 in Text S1), which was shown to outperform Seed-and-Wobble on a set of five TFs in a similar assessment using cross-validation over probe designs. However, even in this assessment, RankMotif++ did not outperform an 8-mer based method similar to E-max. More precisely, instead of using E-scores, 8-mers were scored by their median training probe intensity or “Z-score”, and test probe sequences were scored by the maximal median intensity over 8-mers, which we can call the “Z-max” approach. For completeness, we did a complete benchmarking of the E-max and Z-max methods and found no significant difference in their performance (Figure S2 in Text S1). Moreover, we found no significant difference in performance between RankMotif and Seed-and-Wobble, while we found that SVR models significantly outperformed RankMotif (Figure S3 in Text S1). Therefore, the main conclusion of the previous RankMotif study – namely, that a PSSM method can be competitive with a 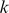-mer scoring derived from simple statistics on the probe intensity data – is consistent with our findings. However, we additionally find that supervised discriminative models with SVRs strongly outperform both PSSMs and 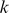-mer scoring for the task of predicting *in vitro* TF binding preferences.

### SVR models improve *in vivo* occupancy prediction in yeast

Next we used the SVR models trained on *in vitro* PBM data to predict *in vivo* occupancy, as measured by chromatin immunoprecipation followed by microarray (ChIP-chip) experiments.

**Figure 3:**
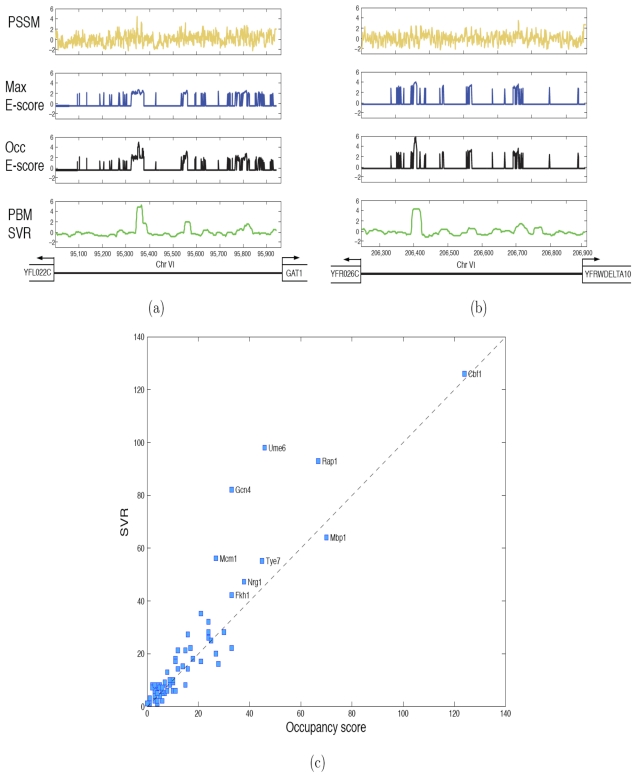
SVRs improve *in vivo* occupancy prediction in yeast. Predicted binding profiles for (a) yeast TF Ume6 along IGR iYFL022C and (b) yeast TF Gal4 along IGR iYFR026C using log-odds ratios for the PBM-derived PSSM motif (gold); max E-score, considering only 8-mer patterns satisfying a minimal E-score threshold of 0.35 (blue); E-score based occupancy, plotting median probe intensity for 8-mer patterns with maximal E-score (black); and SVR prediction scores (green). (c) Scatter plots showing occupancy score predictions (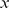-axis) versus SVR (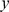-axis) for yeast *in vivo* binding preferences as measured by detection of the top 200 IGRs by the top 200 predictions.

There are 68 yeast TFs for which PBM data and ChIP-chip data are both available. For each TF, we first computed SVR binding profiles along 6724 intergenic regions (IGRs), each 200–2000 nucleotides in length, using a sliding 36-mer window for scoring. Figure 3(a,b) shows predicted binding profiles for two yeast TFs, Ume6 and Gal4, along IGRs that they occupy *in vivo* using different methods: log-odds scores for PBM-derived PSSMs (gold), maximal E-score over a fixed threshold of 0.35 (blue); E-score based occupancy (black), corresponding to the median probe intensity of PBM probes containing the highest-scoring 8-mer pattern [9]; and SVR scores (green). For Ume6, all methods detect this IGR among the top 200 predictions and seem to agree on the location of the highest and second highest peak. For Gal4, the SVR profile and the noisier E-score profiles seem to locate a different binding peak than the PBM-derived PSSM, and only the SVR method detects this IGR among the top 200 predictions. While the ChIP-chip data cannot identify the true location(s) of the binding sites, we do find increasing enrichment for conservation with increasing SVR score (Figure S5 in Text S1), with even moderate scoring peaks showing enrichment for conservation relative to background.

We then compared the performance of SVR models with previously published results based on the E-score occupancy method of [9]. Following the previous analysis, when TF occupancy data is available for more than one condition, we aggregated the data by assigning each IGR the minimal ChIP-chip 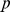-value over conditions (with Bonferroni correction) to obtain as comprehensive a list of true positive IGRs as possible. While [9] used an ROC analysis relative to a fixed 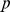-value cut-off of 0.001, we found that AUCs for TFs with very few true positive IGRs were not informative for either method. We instead computed the detection of the top 200 IGRs by the top 200 predictions, where the top 200 “bound” IGRs were determined by their 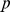-value ranking. For the SVR method, we ranked IGRs by the height of their max peak, while for the E-score occupancy method, we used the scores provided by the authors. Figure 3(c) shows a scatter plot of the detection of the top 200 IGRs by SVR and E-score occupancy.

Since chromatin state and interactions with other DNA-binding factors influence *in vivo* occupancy, we do not expect a TF's sequence signal alone to perfectly correlate with the occupancy data. In fact, similar to the results reported by [9], prediction of *in vivo* occupancy is weak to very poor (fewer than 40 of the top 200 IGRs detected) by both methods for most TFs. However, for the TFs with the best results by the E-score occupancy method (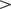
40 top IGRs detected), the SVR method outperforms the previous occupancy score method in 8 out of 9 cases, sometimes to a large degree. [9] performed extensive motif analysis to give evidence that indirect binding may account for a part of the TF occupancy signal in yeast. We too hypothesized that interpreting TF occupancy is confounded by indirect or competitive binding. We performed a detailed analysis of potential interactions between TFs and cooperative or competing partners (see Figure S6 in Text S1), and we found for 26 out of the 68 yeast TFs, the TF's *in vivo* occupancy is well predicted by the SVR model of a second potential “partner” TF (Figure S6 in Text S1).

As we did for the *in vitro* cross-validation experiments, we also benchmarked SVR and E-score occupancy against PBM-derived PSSMs from [9], where we scanned PSSMs across IGR sequences and scored each IGR by its maximum log odds score. Again, we evaluated performance by counting the detection of the top 200 IGRs based in the top 200 predictions, and we found that for the 9 well-predicted TFs, the SVR model outperforms PSSMs (6 wins, 1 ties, 2 losses) while the E-score occupancy performs worse than PSSMs for 5 of these 9 TFs (Figure S7 in Text S1).

### SVR models improve genomic occupancy prediction in mammalian genomes

We next evaluated the performance of PBM-derived SVR models for the prediction of TF occupancy in mouse and human genomes. We examined seven ChIP-seq experiments conducted in three different cell types: Oct4, Sox2, Klf4, and Esrrb in E14 mouse ES cells [14]; Srf and Gabpa in GM12878 cells (human EBV-transformed B-lymphocytes) [15]; and Hnf4a in HepG2 cells [16]. For TFs whose binding domain is not present in the UniPROBE PBM database, we used the most similar binding domain(s) with available PBM data: Pou2f3 and Pou2f1 substituting for Oct4; Sox12 and Sox21 for Sox2; Klf7 for Klf4; and Esrra for Esrrb. Both Pou2f3 and Pou2f1 differ from Oct4 by just one residue in their DNA-contact residues, based on homeodomain-DNA contacts determined from the 3D structure for Engrailed [17], [18]. Sox2 best aligns with Sox21, but we include Sox12 as well to assess the variability of PBM-derived models for TF domains that are thought to bind similar motifs.

We computed the SVR models by carefully selecting the parameters 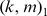 using cross-validation experiments on PBM array data (see Text S1). For our test data, we selected a set of 1000 confident ChIP-seq peak regions and 1000 “negative” regions selected from flanking sequences. More specifically, we extracted 60bp regions centered around the peaks (positive examples) and 60bp regions 300bp away from the peaks (negative examples). Model performance was measured by the area under the ROC curve (AUC), using the maximum SVR prediction score (over 36-mer windows) to rank the ChIP-seq 60-mers. We compared our SVR models to the occupancy score derived from E-scores [9]. We also compared to PSSMs extracted from PBM data with the Seed-and-Wobble algorithm [3], [10], which are available for download from UniPROBE [11].

**Figure 4:**
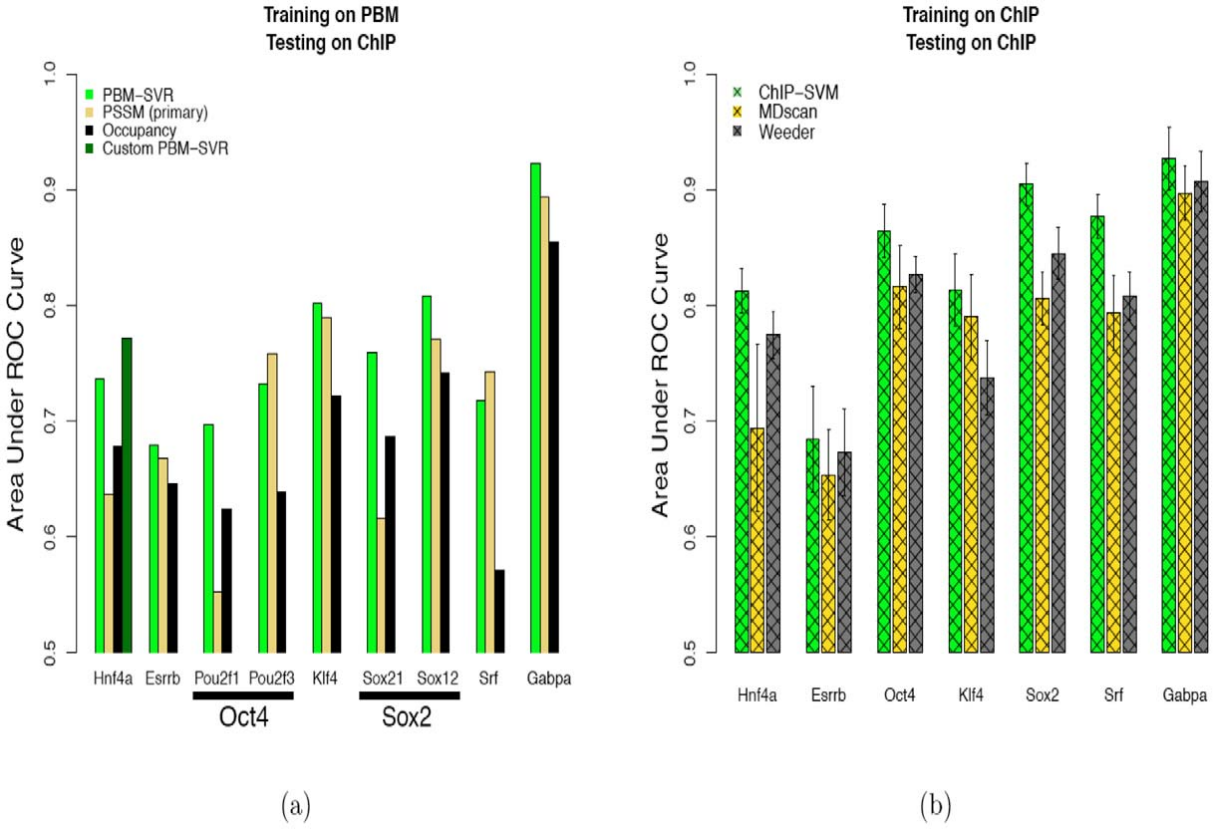
Predicting TF occupancy in mouse and human genomes as evaluated on ChIP-seq data. (a) SVRs trained on PBM arrays are able to capture ChIP-seq peaks better than PSSMs or the occupancy score. (b) SVMs trained on ChIP-seq data capture sequence information from the genomic context of ChIP-seq peaks and improve *in vivo* prediction performance.

Figure 4(a) shows AUC results for all three methods; here, in cases where UniPROBE reports both primary and secondary PSSMs, we show results for the primary motif. We found that SVR outperforms both the PSSM and occupancy score methods in 7 out of 9 cases, where we report results for models trained on two different PBM experiments to predict Oct4 and Sox2 occupancy. There were two TFs, Sox21 and Hnf4a, for which the secondary PSSM outperformed the primary PSSM. However, in each case, the improved AUC (0.75 and 0.70, respectively) was still worse than the performance of the PBM-derived SVR.

We report two SVR results for Hnf4a, one using the mouse PBM array data present in the UniPROBE database (Figure 4(a), Hnf4a, leftmost bar), the other using a novel PBM array design developed specifically for human Hnf4a and using the purified full-length protein instead of the DNA-binding domain in the PBM experiment [19]. In the latter dataset, short probe sequences were designed based on known motifs for Hnf4a (see Figure S8 in Text S1 for contrasting binding profiles of the standard versus custom PBM array). [19] published two such PBM array designs and, by combining data from the two arrays and modifying our algorithm to accommodate the 13-mers that comprise this PBM data, we were able to train an SVR model that gave the best predictions of Hnf4a *in vivo* binding (Figure 4(a), Hnf4a, rightmost bar).

### Training discriminative models directly on ChIP-seq data improves occupancy prediction

Since PBM arrays are limited to capturing the *in vitro* binding preferences of transcription factor domains, we hypothesized that additional sequence signals may be present in ChIP-seq data and enable improved prediction performance. We therefore trained support vector machines (SVMs) using the standard 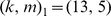 parameters on 60-mer ChIP-seq peaks (positive sequences) and flanking negative sequences. This training procedure potentially allows the SVM to capture sequence information for both the chromatin immunoprecipitated TF and its *cis* cofactors. We evaluated performance by computing AUCs on the same test sets of 1000 ChIP-seq peaks and 1000 flanking negative sequences using 10-fold cross-validation.

For a method comparison, we used two popular motif discovery algorithms, Weeder [7] and MDscan [8], which determine overrepresented 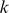-mer and PSSM motifs, respectively. Again, we tested these methods using 10-fold cross-validation and evaluating AUCs on held-out folds.

Weeder performs an exhaustive search for the most overrepresented 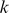-mer patterns for a given specified size 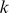. We used the algorithm to find the top 50 enriched motifs in the training data, allowing up to one mismatch. To make predictions, we counted the occurrences of these motifs in the test sequences, again allowing up to one mismatch, and used this count to rank the test sequences. We tested 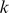-mer lengths 6, 8 and 10, and reported results for the best performing model. MDscan identifies overrepresented motifs by iteratively constructing PSSMs and using binding site flanking regions to define a Markov chain background model. We applied the highest scoring PSSM as found by MDscan to the test sequences, using a zero order Markov model based on nucleotide frequencies in the human genome as the background model. (We did not use a first order Markov background model since we found it slightly decreased PSSM performance for all but one TF.) We experimented with motif lengths of 8, 10 12, 14 and 16 and reported the best results.

Figure 4(b) shows results for ChIP-derived SVM models and the motif discovery approaches for the occupancy prediction task. We first note that for 4 out of 6 TFs, the ChIP-derived SVM model significantly outperforms the corresponding PBM-derived SVR model(s). The exceptions are Essrb and Gabpa, where there is little difference in performance between the *in vitro* and *in vivo* models. Furthermore, although Weeder and MDscan yielded predictions with AUCs above 0.65 for all 7 TFs, the SVM model outperformed both methods in every case that we considered (Figure 4(b)), sometimes by more than 0.1 in the AUC score. It is also worth noting that while Weeder and MDscan required parameter tuning, the SVM model parameters were kept fixed. As a final method comparison, we also tested a newer motif discovery algorithm called cERMIT [20] on these data sets, and we again found that the SVM models outperformed the best-performing PSSM returned by cERMIT (Figure S11 in Text S1).

We caution that our sample set of TFs is small: although we did a thorough search for all available ChIP-seq data sets, we found only a small number of TFs with both PBM and ChIP-seq data; moreover, in some cases, the TF domain represented in the PBM experiment is slightly different than in the TF ChIP-seq experiment. It will therefore be important to repeat these experiments on a wider range of TFs once suitable data becomes available.

Finally, we evaluated whether there was any advantage to training regression models on ChIP-seq peaks labeled with real-valued occupancy rather that binary classifiers to discriminate between peaks/non-peaks. We found that SVR models trained with real-valued labels gave slightly worse performance in our AUC analysis as compared to SVM models (see Figure S11 in Text S1). We hypothesize that either (i) the currently available ChIP-seq derived occupancy scores are not yet quantitative enough to use to train a regression model or (ii) the best predictor of peak height/occupancy score is not the sequence signal itself but chromatin state (accessibility of the DNA, nucleosome positioning, histone modifications).

### PBM experiments may capture alternate motifs that are not preferred *in vivo*

To understand the differences between *in vitro* and *in vivo* TF binding models, we developed an approach to examine the sequence information extracted by the SVR/SVM models. It is of course possible to simply examine the top-weighted 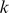-mers in the SVR/SVM weight vectors; for example, the 13-mers with highest positive weights in the PBM-derived SVR models often contain subsequences that resemble the Seed-and-Wobble motifs derived from the same data (Tables S2 and S3 in Text S1). We sought instead to visualize the full 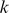-mer content of the model. We first looked at the *in vitro* models for Oct4, since the PBM-derived PSSMs for the two selected “nearest neighbor” Pou domains had very different performance, and we wanted to understand the source of the instability.

We used a feature analysis procedure to look inside the “black box” of the PBM-derived SVR model for Pou2f3, the neighbor of Oct4 with the better performing PSSM. The solution of the SVR optimization problem determines a weight vector 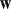 over the space of 13-mer sequence features; 13-mers with high weights contribute the most to high binding prediction scores. The basic idea is to represent the similarity of 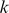-mer features based on their support across the training data and also visually represent the weight of the features in the SVR/SVM model. In this way, we avoid doing too much *post hoc* summarization of the 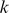-mers, and instead we represent the features more as they are used and contribute to the model.

**Figure 5:**
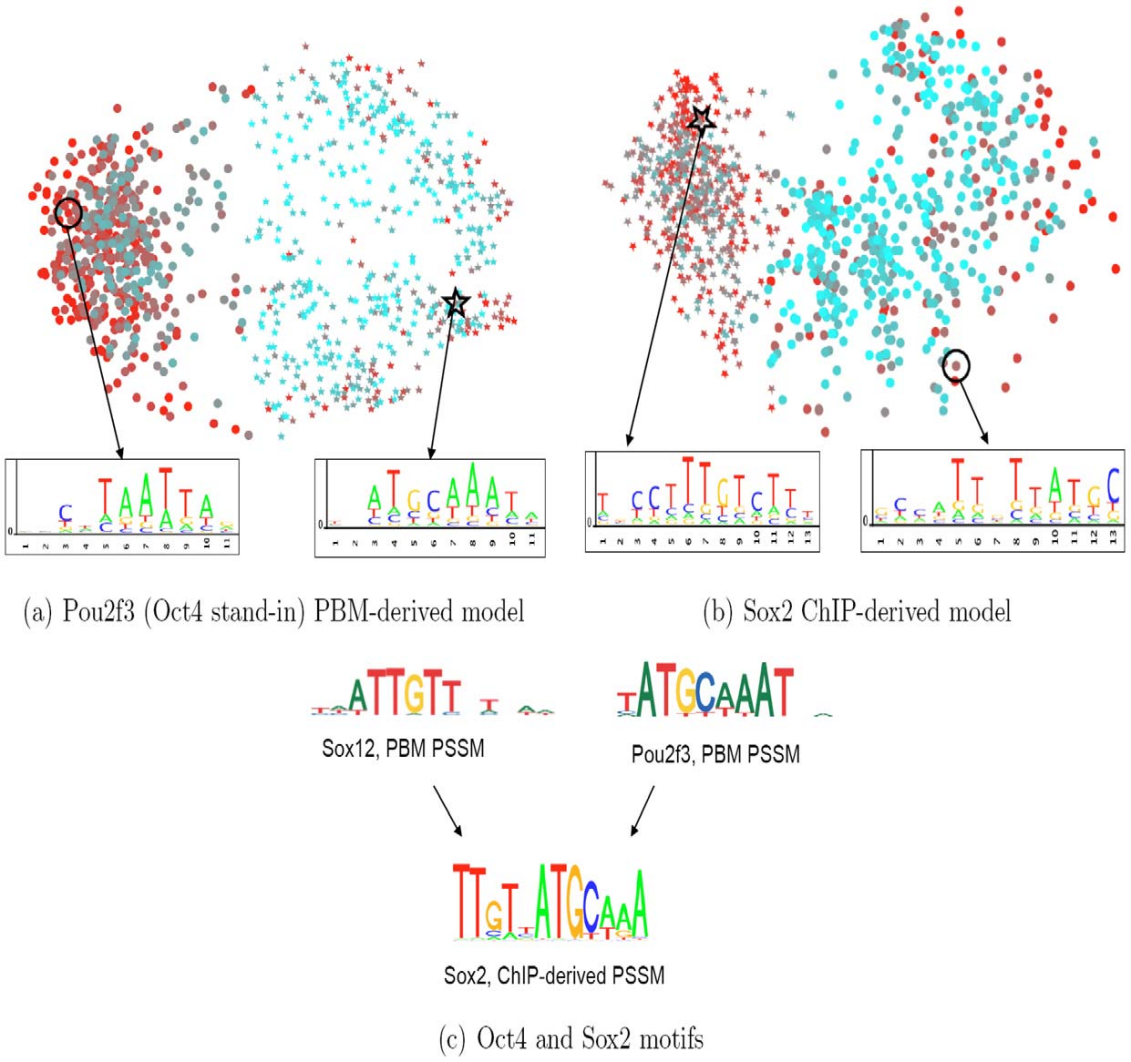
Sequence feature analysis of *in vitro* and *in vivo* models. We plot 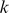-mers contributing to the (a) Oct4 PBM model and (b) Sox2 ChIP model, where each point represents a 13-mer and is colored according to its model weight (red for high weights, blue for low weights). Star and circle point styles indicate different clusters. For the PBM-derived model, the clusters appear to represent primary and secondary binding motifs, with the more degenerate motif perhaps arising as an artifact of the PBM experiment. For the ChIP-derived model, the clusters correspond to the motifs for Sox2 and its cofactor Oct4. (c) PBM-derived PSSMs for Sox12 and Pou2f3, downloaded from UniPROBE, and ChIP-derived PSSM for Sox2, computed using MDscan on the Sox2 ChIP-peak sequences (60bp long).

To obtain a similarity measure between these features, we represented each 13-mer by the vector of its alignment scores to the training sequences (see Materials and Methods). Intuitively, 13-mers that are close in Hamming distance will be represented by nearby vectors in this representation. After clustering 13-mer features based on this vector representation and projecting to a two-dimensional representation (see Materials and Methods), we identified two clusters of features, shown in Figure 5(a) using stars and circles. The color scheme indicates the SVR weight associated with the 13-mer feature, red for highly weighted features and blue for low weights. The two well-separated clusters suggest that the SVR is learning a primary and secondary motif, similar to results of PSSM-based analysis [10]. We took a 13-mer feature near the centroid of each cluster and expanded each into a PSSM by aligning to the positive training sequences (see Materials and Methods): the “star” cluster is represented by a motif that looks like the canonical Oct4 octamer (ATGCAAAT), but the “circle” cluster is centered on a more degenerate (TAATT) motif. To determine the *in vivo* prediction performance of each cluster independently, we retrained SVR models using the star and circle 13-mer features separately and obtained dramatically different AUCs of 0.75 and 0.54, respectively, on the Oct4 ChIP-seq data. The poor *in vivo* performance of the star cluster of features suggests that the PBM is learning a secondary motif that is not preferred *in vivo*. The presence of these apparently PBM-specific features only slightly degrades the performance of the full SVR model (AUC of.74) but may seriously impact PSSM-based methods. For example, the Seed-and-Wobble algorithm identifies a primary motif similar to TAATTA for the other Oct4 nearest neighbor, Pou2f1 (see Figure S10 in Text S1), which accounts for its poor occupancy prediction. We reiterate the caveat that neither of these Pou domains is in fact Oct4; it is conceivable that the differences between PBM and ChIP binding preferences are due in part to differences in these homeodomains.

### ChIP-derived SVMs capture information about cofactor motifs

We next performed a similar feature analysis of the ChIP-derived model for Sox2, one of the examples where the *in vivo* model strongly outperformed the *in vitro* model. Here, 13-mer features from the SVM model are represented by their vector of alignment scores relative to 60bp sequences under ChIP-seq peaks rather than probe sequences.

Again, we identified two well-separated clusters, shown using stars and circles in Figure 5(b). Here, the cluster representative for the “star” cluster can be expanded to a PSSM that closely resembles the Sox2 motif. However, the representative for the “circle” cluster maps to part of the Oct4 octamer motif, indicating that the ChIP-derived model is learning binding information about Sox2's binding partner Oct4 (Figure 5(c)). We hypothesized that this additional *cis* information may account for part of the improvement of the *in vivo* model over the PBM-derived model. To quantify this effect, we identified Sox2 bound regions that are not detected by the PBM-trained SVR model but are correctly detected by the ChIP-trained SVM (Figure S9(a) in Text S1). These 33 60bp regions were 6-fold depleted for the core Sox2 motif TTGT and 3-fold enriched for the core Oct4 motif TGCA. Moreover, 32 out of 33 of these regions were detected as positives by the PBM-trained SVR for Oct4 (). These results suggest that some binding of Sox2 may be indirect via binding of the cofactor Oct4.

## Discussion

We have presented a flexible new discriminative framework for learning TF binding models from high resolution *in vitro* and *in vivo* data. In particular, we showed that SVR models using string kernels outperform existing existing approaches like PSSMs and E-scores for predicting *in vitro* TF binding preferences as measured by PBM experiments, based on cross-validation experiments across array designs. We also found that PBM-derived SVR models improve *in vivo* occupancy prediction over PBM-derived PSSMs and E-scores, in particular when ChIP-seq (as opposed to lower resolution ChIP-chip) data is available for validation. Furthermore, we saw that by training directly on ChIP-seq, i.e. using ChIP-seq peaks to define positive genomic training sequences and taking non-peak regions as negative sequences, we can significantly improve over PBM-derived models and outperform existing motif discovery methods. We also described a feature analysis procedure for looking inside the “black box” of the trained SVR/SVM models to identify clusters of sequence features that contribute to binding predictions. Importantly, this analysis allowed us to confirm that ChIP-trained SVM models were learning additional sequence signals corresponding to cofactor binding sites.

PSSMs have a long history in the analysis of TF binding sites and remain ubiquitous due to their interpretability. However, as we continue to accumulate mammalian PBM data and ChIP-seq data, the more general models that we develop here—i.e. models that do not force a PSSM representation on binding sites and can integrate *in vivo* sequence signals from both a TF and its cofactors—may be more suitable for representing complex regulatory regions. We anticipate a number of directions for building on this work. First, we can develop strategies to train jointly on PBM and ChIP-seq data for the same TF in order to cleanly disambiguate between direct and indirect binding. Second, as more PBM data becomes available, we can develop multi-task training strategies for modeling the binding preferences of a class of structurally related TFs, using features of the amino acid sequence as well as a 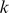-mer representation of probe sequences. Then, given a new TF for which PBM data is not available, the model would extend to predict its binding preferences. Third, we can combine our *in vivo* TF sequence preference models with data on chromatin state, including histone modifications and DNase I footprinting, using a kernel combination strategy. The goal would be to predict TF target genes in new cell types, given only the chromatin information in the cell type, after training on ChIP-seq data paired with chromatin data in other cell types. Therefore, the flexible sequence-based framework we describe here provides the foundation for the systematic modeling of genome-wide TF occupancy.

## Materials and Methods

### Overview of SVR training

We developed a training strategy for our SVR models that involved three key components: (1) the choice of kernel, which specifies the space of features used to compare pairs of probe sequences; (2) the sampling procedure for selecting the training sequences, which produces more informative training data and reduces training time; (3) the feature selection method, which eliminates unimportant features and further improves computational efficiency. Each component is described in more technical detail below. We used the LIBSVM package for the computation of SVR models, keeping the 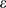 parameter fixed at 0.1 for all experiments.

### The di-mismatch kernel

Training a kernel method like an SVR on sequence data requires the use of some kind of string kernel, i.e. a similarity measure between sequences that defines an inner product in a corresponding feature space. Various 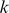-mer based string kernels have been proposed, including the 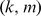 mismatch kernel [5], where the feature representation for a sequence amounts to an inexact-matching histogram of 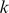-mer counts, allowing up to 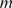 mismatches in each 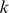-mer match (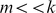). Here, however, even for small values of the mismatch parameter 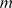, this kernel tends to make the “mismatch” neighborhood of a given 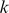-mer too large.

We therefore developed a novel first order Markov mismatch kernel, called the *di*-mismatch kernel, that counts mismatching dinucleotides and that inherently favors 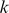-mers with consecutive mismatches. Let 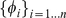 be a set of unique 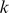-mers that occur in the set of training sequences (PBM probe sequences). Given a training sequence 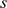 of length 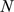, we define the set of substrings of length 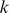 in 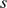 to be
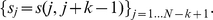
Then 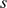 may be represented by the feature vector
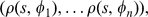
where 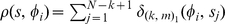, and the value 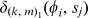 is the di-mismatch score between two 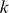-mers, which counts the number of matching dinucleotides between 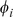 and 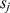, that number being set to zero if this count falls below the threshold 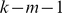, where 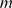 is the maximum number of mismatches allowed.

This score inherently favors consecutive mismatches, as we show in the following examples. Consider the first pair of 13-mers shown with four non-consecutive mismatches, which results in 6 mismatching dinucleotides out of 12:
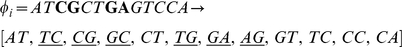


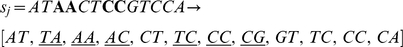
In contrast, the following pair of 13-mers with four consecutive mismatches leads to a count of 5 mismatching dinucleotides.
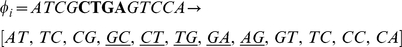


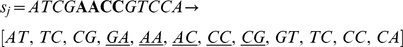



By enforcing a mismatch parameter of 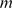, we induce sparsity in the feature counts and seem to obtain more meaningful “neighborhoods” of the features 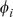 than the standard mismatch kernel. This procedure appeared to capture the full dynamic range of effective binding while downsampling the large number of unbound probes.

Since the PBM arrays are designed to give good coverage of 8-mer patterns (including gapped patterns), we chose 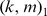 parameters that would require at least 8 matching characters between the 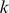-mers. Our parameter experiments on one set of yeast PBM arrays [9] indicated 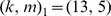 to be the best parameter setting, and we used this kernel choice for the *in vitro* evaluation for most of our reported results. However, one may use a 10-fold cross-validation approach on the training PBM array to perform a grid-search and thereby optimize the choice of the 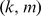 parameters. We used such a strategy for the 7 mammalian *in vivo* occupancy predictions (Figure 4), whereby we tested 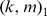 parameters ranging from 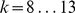 and 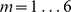 where 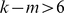.

Much like the mismatch kernel, the computational cost of scoring test sequences with the trained di-mismatch SVM/SVR model is linear with respect to the input sequence length. Every 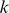-mer has a non-zero match score to a fixed number of features, and each feature is represented by a weight in the support vector model. Therefore, the contribution of each 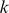-mer can be pre-computed, and those with non-zero contribution can be stored in a hash table.

### Sampling PBM data to obtain an informative training set

Standard PBM arrays typically contain 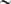
44K probes, each associated with a binding intensity score, but only few hundred probes indicate some level of TF binding. Using all of the PBM probes as training data would allow the SVR to achieve good training loss simply by learning that most probes have low binding scores. In order to learn sequence information associated with the bound probes, we selected training sequences from the tails of the distribution of the normalized binding intensities. More specifically, we selected the set of “positive” training probes to be those sequences associated with normalized binding intensities 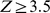; if the number of such probes was less than 500, we selected the top 500 probes ranked by their binding signals. The same number of “negative” training probes was selected from the other end of the distribution. This procedure appeared to capture the full dynamic range of effective binding for learning the regression model while downsampling the large number of unbound probes. We also tried sampling “negative” probes from the full intensity distribution (anywhere outside the positive tail), but we found that using the negative tail yielded better results.

### Feature selection

Careful feature selection can eliminate noisy features and of course reduces computational costs, both in the training and testing of the model. In particular, choosing very infrequent 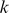-mers may add noise, and ideally, sequence features should display a preference either for bound or unbound probes. Therefore, we selected the feature set 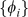 to be those 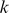-mers that are over-represented either in the “positive” or “negative” probe class by computing the mean di-mismatch score for each 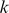-mer in each class and ranking features by the difference between these means. In all reported results, we used at most 4000 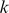-mers for our models.

### ChIP-seq processing

We processed the ChIP-seq data using the SPP package [21] and extracted the top 1000 peaks to define a gold standard for occupancy. A 60bp window was selected around each of the peaks and used as the positive examples. A 60bp window 300bp to the left of the peak was selected as the negative example.

### Extracting features from SVR/SVM models

It is informative to be able to use the SVM model to visualize the 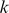-mers that contribute to the model. Here our goal is to visually represent both (i) the similarity of 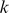-mer features based on their support across the training data representing and (ii) the contribution (or weight) of these 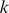-mers to the model.

To obtain a similarity measure between 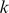-mer features extracted from the trained models, we represented each 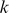-mer by a vector of alignment scores against the positive training sequences used to compute the SVR model: we found the optimal ungapped alignment of the 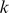-mer to each training sequence and used the number of match positions as the alignment score. Intuitively, sequence-similar 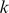-mers will have similar alignment scores across the training examples, so they will be represented by nearby vectors in this representation. However, we are not explicitly modeling sequence dependence but instead relying on co-occurrence of matches of similar 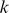-mers. We then performed 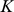-means clustering (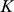 = 2) on the vectors representing the 13-mer features. Next we used the SVM/SVR weight vector 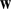, derived from the solution to the optimization problem, to select the top 500 representatives for each cluster, thereby reducing the rest of our analysis to 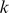-mers that contributed significantly to the model. Next, we projected the 1000 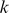-mers to a two-dimensional representation using principal component analysis (PCA), distinguishing cluster members with circles and stars in the plot. The relative significance of each feature is indicated by a color scale ranging from red to blue, for high and low 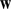 respectively.

Finally, we defined a cluster representative for each group by the feature that has the following two properties: (i) it is in the top quartile of the 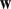 weights for that cluster, and (ii) it is the closest feature to the cluster centroid. This gives us a cluster representative that is simultaneously close to the true cluster centroid and significant for the model. Finally, to represent a given 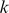-mer feature by a motif logo, we selected the top 50 positive training sequences that best aligned with the 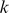-mer, extracted the 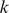-length sequences that matched the feature, and computed a PSSM.

## Supporting Information

Text S1 High resolution models of transcription factor-DNA affinities improve in vitro and in vivo binding predictions: Supplementary information.
(1.13 MB PDF)
