# Supplementary material for: High Resolution Models of Transcription Factor-DNA Affinities Improve In Vitro and In Vivo Binding Predictions
Source: PLoS Comput Biol. 2010 Sep 9;6(9):e1000916. doi: 10.1371/journal.pcbi.1000916 (PMC2936517; doi:10.1371/journal.pcbi.1000916)
Supplement: Text S1 — High resolution models of transcription factor-DNA affinities improve in vitro and in vivo binding predictions: Supplementary information. (1.13 MB PDF) [file pcbi.1000916.s001.pdf]

High resolution models of transcription factor-DNA affinities improve *in vitro* and *in vivo* binding predictions: supplementary methods

Phaedra Agius[1], Aaron Arvey[1], William Chang[1], William Stafford Noble[2], Christina Leslie[1*]
[1]Computational Biology Program, Memorial Sloan-Kettering Cancer Center, New York, NY
[2]Department of Genome Sciences, University of Washington, Seattle, WA

August 2, 2010

Comparison of SVR, E-scores, and PSSMs on *in vitro* data

We previously showed that SVR *in vitro* predictions outperformed the maximum E-score approach (E-max) across yeast and mouse TFs (see Results, Figure 2). Supplementary Figure 1 shows that (a) PBM-derived PSSMs and E-max give comparable performance in cross-validation on probe designs, while (b) PBM-trained SVR models clearly outperform PSSMs.

In Figure 2, we compare the performance obtained by scoring PBM probe sequences using the maximum E-scores ("E-max" method) or Z-scores ("Z-max" method) over constituent k-mer patterns, where Z-scores are the median of the transformed probe intensities (see Badis et al. (2009)). As in the paper, we evaluated performance on TFs for which PBMs experiments on two array designs are available, using E-scores or Z-scores estimated on one array to make predictions for the other array. Using the detection of the top 100 as our evaluation measure, we found no statistically significant performance difference between Emax and Zmax (Emax is equivalent or superior to Zmax in 68 out of 114 cases, and the mean score for each is 20.7 and 20.3 respectively). A signed rank test shows no significant improvement of one method over the other for the detection of the top 100 probes using a significance threshold of $p < 0.05$. These results indicate that either E-max or Z-max can be used as essentially equivalent baseline methods. We chose the E-max approach since E-scores were readily available for all the data at the time of processing.

To examine the choice of Seed-n-Wobble as our baseline method for deriving PSSMs from PBM experiments, we ran the RankMotif algorithm on PBM data for mouse TFs for which two array designs are available. As before, we learned PSSMs on one array and tested on the other. Since RankMotif requires selection of a motif length parameter, we tested lengths 6-13, and we chose the length with the highest likelihood for each PBM array using only the training data. We also constrained our search to PSSMs of length 10 to determine the accuracy of a single parameter setting.

Figure 3 shows the comparison of the performance of Seed-n-Wobble PSSMs, SVR models, and RankMotif. We found that Seed-n-Wobble slightly outperforms RankMotif when a fixed length of 10bp is used, whereas RankMotif slightly outperforms Seed-n-Wobble when using the optimal (on

*Corresponding author, cleslie@cbio.mskcc.org

the trainig set) PSSM length, measured in terms of wins/losses over PBM experiments. However, neither of these performance differences is statistically significant at a p-value threshold of 0.05 (signed rank test). Meanwhile, the SVR model significantly outperforms RankMotif whether the optimized length or fixed length 10 is used in the RankMotif approach ($p < 3.7e-05$ and $p < 3.4e-05$, respectively, signed rank test).

Alternative kernel methods

When developing our approach, we started by testing the spectrum and mismatch kernels. However, our initial experiments using these existing kernels to train SVR models showed little improvement over the E-max approach, which prompted our search for a more effective string kernel for this particular problem. In Figure 4, we compare the di-mismatch kernel against the regular mismatch kernel for $(k, m) = (8, 0)$, i.e. 8-mers with no mismatches, and $(k, m) = (9, 1)$, i.e. 9-mers with one mismatch, using the same cross-validation approach as in the paper (train on one PBM array design, test on the other). The di-mismatch kernel has equal or better performance than the 8-mer kernel on 30 out of 33 yeast TFs and 70 out of 115 mouse TFs. Similarly, the di-mismatch kernel equals or outperforms the regular $(9, 1)$ mismatch kernel for 22 out of 30 yeast TFs, and 62 out of 115 mouse TFs. While the advantage of the di-mismatch kernel seems at first more marginal for the mouse TF data set, we do see a trend where the regular mismatch kernel tends to win only on poorly performing examples (i.e. TFs where both kernels detect < 20 true positives), while the di-mismatch kernel tends to win on the more successful examples. More specifically, out of the 52 cases where di-mismatch and 8-mers detect more than 20 of the top 100, the di-mismatch approach outperforms 8mers on 47 cases, with 2 ties. Similarly, di-mismatch outperforms the (9,1)-mismatch kernel ("1-gapped 8-mers") on 41 out of the 75 best cases (with 8 ties). We also find that the $(13, 5)$-di-mismatch kernel convincingly outperforms the $(13, 5)$-mismatch kernel, suggesting that in the regular nucleotide alphabet, allowing 5 mismatches is too permissive. Note that the mismatch kernel results shown in Figure 4 involved the same feature selection as we used for our di-mismatch SVR model. We ran experiments to see whether the feature selection hurt the mismatch model and found no such evidence.

Conservation and SVR occupancy profiles

Traditional motif analysis uses conservation as a filter to enrich for functional binding sites. Instead, we examined the extent to which peaks in the SVR-predicted occupancy profiles already coincide with conserved regions. For each yeast TF, we Z-normalized the SVR peaks obtained for all the IGRs, and we partitioned these into occupancy bins of 0-0.5, 0.5-2, 2-6 and >6 standard deviations, each bin corresponding to predictions of background, weak binding, moderate binding, and strong binding regions, respectively. For each peak, we defined the "flank sequences" to be those regions flanking the peak that have SVR scores within the range specified by the bin to which the peak is assigned. We downloaded phastCons (Siepel et al., 2005) yeast conservation scores based on the multiple alignment of seven Saccharomyces genomes (paradoxus, mikatae, kudriavzevii, bayanus, castelli, kluyveri) for yeast IGR sequences from the UCSC Genome Browser database. Finally, we assigned a conservation score to each SVR peak by mapping the flank sequences to our downloaded conservation scores, and we aggregated all peak conservation scores over the 68 yeast TFs that we considered. The cumulative distributions of these phastCons conservation levels indicate increased conservation as the SVR peaks increase. Interestingly, we also observe some conservation for the

"moderate binding" bin.

Cooperative and competing factors may help explain TF occupancy in yeast

One confounding issue in the interpretation of TF occupancy data is the interaction of the immunoprecipitated TF with other DNA-binding proteins. A previous analysis of yeast occupancy data sought to distinguish between direct and indirect TF binding by looking for enrichment of PBM-derived motifs for other factors in a TF's ChIP chip signal for a given condition (Gordân et al., 2009). In particular, if the PBM-derived motif of a *base TF* B was not enriched in B-occupied IGRs, but the motif for a *partner TF* P was enriched, then P was proposed as a potential binding partner for B. According to this model, B would tend to bind its IGRs indirectly via interaction with its partner P, while P would directly bind sites in these regions.

Motivated by this analysis, we examined all pairs of TFs (B, P) where the SVR model for partner P was able to significantly predict the *in vivo* occupancy of B. We limited our analysis to the rich media (YPD) condition, for which the most occupancy data was available. We performed an AUC analysis where we took true positives to be B-occupied IGRs satisfying a p-value threshold of 0.01 and true negatives to be IGRs assigned a p-value greater than 0.5. We use a more relaxed $p < 0.01$ threshold for occupancy in order to avoid statistical issues with very small numbers of positives in the AUC analysis. We then identified all TFs P such that AUC performance of P's SVR model for predicting occupancy by B, denoted as $f_P(B)$, exceeds the best performance of randomized TF SVR models. More specifically, a randomized TF SVR model refers to the AUC obtained when the SVR of TF P_i is applied to randomly perturbed IGR sequences and validated by B's true binding preferences - we denote this as $f_{P_i}(B_{rand})$. We further considered whether TFs B and P had similar (a) true occupancy and/or (b) predicted occupancy, both being based on the overlap of true/predicted occupied IGRs, using Fisher's exact test to determine appropriate p-values, with $p < 0.05$ suggesting significant overlap.

Similar to the PBM-derived motif enrichment analysis of Gordân et al. (2009), we found many TFs B (26 out of 68) that could be paired with at least one parnter P whose SVR models significantly predicted B-occupied IGRs in rich media conditions (see Figure 6). Of these, we found that 22 TFs had at least one partner P whose true occupancy was similar to B's true occupancy. When we found pairs of TFs B and P whose predicted occupancy profiles were similar, it was generally true that their PBM-derived motifs were almost identical, suggesting that the TFs prefer to bind the same sites and that they may compete for these sites *in vivo*. As examples, we found that the pair of TFs Cbf1 and Tye7, which have near identical PBM-derived motifs and similar predicted occupancy patterns, each predicts the other's true occupied IGRs. Similarly, the TFs Fhk1 and Fhk2 have similar predicted occupancies and similar motifs, and each predicts the other's occupancy. These potentially interacting pairs were also identified by Gordân et al. (2009), though Fhk1 and Fhk2 are considered to have distinct motifs in that study. We also found that the Rap1 SVR model significantly predicted occupancy for Fhl1, Gat3, and Pdr1; only the first of these potential interactions was reported in the previous study.

Comparison of SVR, E-scores, and PSSMs on yeast *in vivo* data

In Results, Figure 3 we compared SVR *in vivo* predictions with the E-score occupancy method for 68 yeast TFs, and we found that SVR significantly outperformed E-score occupancy on TFs where performance was relatively good for both methods. Supplementary Figure 7 shows that (a) PSSMs

perform slightly better than the E-score occupancy, but (b) SVR outperforms PSSMs, where the advantage is visible forTFs with a detection of at least 40 bound IGRs in the top 200 predictions.

Training on custom PBM array designs

Our SVR model can accommodate training sequences of any length. Here we trained an *in vitro* binding model using data from a custom PBM array designed for the human liver-specific TF Hnf4a (Bolotin et al., 2009). For this PBM, Bolotin et al. designed an initial PBM array (PBM1) using probe sequences similar to known binding motifs for Hnf4a; they then trained an SVM to make predictions of possible additional binding site sequences for the design of a second array (PBM2). Each array comprised about 3000 probe sequences (including random controls) of length 13. Using both PBMs, we trained a SVR model and tested it on the ChIP-seq to obtain an AUC of 0.77, a slight improvement over the AUC of 0.74 obtained using the mouse 36-mer PBM array from (Badis et al., 2009) (see Results, Figure 4(a)).

Figure 8 shows predicted binding profiles for the two models at two ChIP-seq peaks where we see that the 13-mer model has a visibly more peaky signal (dark green) than the standard 36-mer model (light green).

Predicting TF binding in mammalian genomes

As described in the main text, our SVR models for the mammalian ChIP-seq data were trained on the PBM arrays using optimized $(k, m)_1$ parameters for the di-mismatch kernel. Specifically, we performed 5-fold cross-validation on the PBM arrays using a grid search to select k and m. Table 1 shows the optimal $(k, m)_1$ parameters for the 7 mammalian examples that we considered.

**TF**	k	m
Hnf4a	9	2
Pou2f3	11	2
Klf7	13	4
Srf	12	4
Esrra	10	2
Gabpa	11	3
Sox12	9	1

Table 1: Parameter choices based on cross-validation on PBM data.

We found that the predictions for Oct4 and Sox2 ChIP-seq peaks depended on which neighbor was used for the PBM array. The canonical motif for both Pou2f1 and Pou2f3 is the octamer ATGCAAAT. However, the Seed-and-Wobble algorithm, which uses the 8-mer pattern with the highest E-score as a seed, identifies TAATTA for Pou2f1 and ATGCAAT for Pou2f3. Thus the PSSM results for Oct4, which are highly dependent on the seed and specific domain chosen, varied considerably from one neighbor to another. On the other hand the SVR approach, which includes a wider selection of k-mers, shows less variability from one neighbor to another. We also observed some overlap between the top k-mers for Pou2f1 and Sox21, the highest E-scoring k-mers being A.A.TAATTA and ATTATAAT, respectively. Neither k-mer has been identified as a binding motif

for the Oct or Sox protein families. The presence of these AT-rich motifs may be attributable to an unknown bias of PBM experiments, but the exact cause remains to be identified.

In an effort to understand the differences between the PBM-trained SVR and the ChIP-trained SVM for Oct4 and Sox2, we took a closer look at the classification and the ranking of the ChIP-seq test sequences as defined by each model. We found a good number of bound regions that are detectable by ChIP-derived SVM but not by PBM-derived SVR for both TFs; these are highlighted by the black circle in Figure 9. For Sox2, we found these regions to be 6-fold depleted for the Sox2 core motif TTGT and 3-fold enriched for the Oct4 core motif TGCA. This shows that the ChIP-derived SVM is able to detect the Oct4-Sox2 indirect binding patterns that are necessarily missed by the PBM array.

Visualizing the Pou2f3 model in contrast to Pou2f1

In Figure 5 in the paper, we showed the visualization of k-mers for the Pou2f3 PBM model as an Oct4 stand-in. Here we consider the other Pou domain that is also discussed in the paper (see Figure 4), namely Pou2f1. We show a feature extraction for Pou2f1 in Figure 10; we find that the distinction between the octamer and AT-rich sequences is not as clear in Pou2f1 as for Pou2f3: the AT-rich motif (TTAAT) seems to occur in both k-mer clusters. Also, the motif shown on the right looks less like an octamer that the one found for Pou2f3.

Other methods for predicting TF binding in mammalian genomes

Our results in Figure 4 of the paper compare our SVM approach with two popular motif discovery algorithms: Weeder and MDscan. Here tested another, newer motif discovery algorithm called cERMIT Georgiev et al. (2010) for the task of discriminating between mammalian ChIP-seq peaks/non-peaks. Figure 11 shows the best performing cERMIT PSSM for each TF.

We also evaluated whether SVR models trained to predict real-valued outputs (ChIP-seq peaks labeled with a real-valued occupancy) outperformed SVM models trained with binary outputs (peaks vs. non-peaks). Figure 11 also shows SVR results where we performed regression on the log-transform of the MTC method from the SPP package Kharchenko et al. (2008); we find similar but slightly worse performance than the SVM models.

Looking at the top weighted SVR k-mers

In Tables 2 and 3 we show the first five k-mers (and their reverse compliments) as ranked by their weights in the SVR models computed on the PBM data for a selection of yeast and mouse TFs. For comparison, we also show the motifs reported by Zhu et al. (2009) and Badis et al. (2009). By inspection, it is clear that the top SVR k-mers from contain subsequences similar to the motifs.

TF	SVR k-mers		PSSM
Ume6	TTTAACCGCCGAA	TTCGGCGGTTAAA	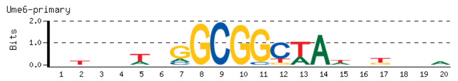
	TTAACCGCGGTTA	TAACCGCGGTTAA	
	CTCGGCGGCTAAC	GTTAGCCGCCGAG	
	ATTAACCGCCGAC	GTCGGCGGTTAAT	
	CTAAGCGCTTAAT	ATTAAGCGCTTAG	
Cbf1	CGTCACGTGACCA	TGGTCACGTGACG	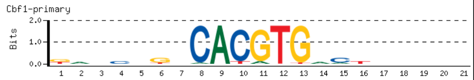
	GGTCACGTGACTA	TAGTCACGTGACC	
	AGGTCACGTGACT	AGTCACGTGACCT	
	GTCACGTGACCAT	ATGGTCACGTGAC	
	GAGGTCACGTGAC	GTCACGTGACCTC	
Rap1	AAGGGTGTACGGA	TCCGTACACCCTT	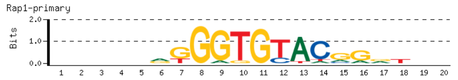
	TCCGTACACCCAA	TTGGGTGTACGGA	
	ACCCCCGTACACC	GGTGTACGGGGGT	
	TTCCGTACACCCA	TGGGTGTACGGAA	
	CAAGGGTGTACGG	CCGTACACCCTTG	
Gcn4	GATGACTCATACC	GGTATGAGTCATC	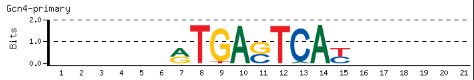
	ATGATGACTCATA	TATGAGTCATCAT	
	TATGAGTCATATT	AATATGACTCATA	
	GTGATGACTCATA	TATGAGTCATCAC	
	GAGTATGACTCAT	ATGAGTCATACTC	
Mbp1	TTACGCGTCGCGT	ACGCGACGCGTAA	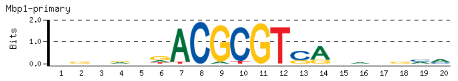
	ACGCGTCGCGTAG	CTACGCGACGCGT	
	TACGCGTCGCGTA	TACGCGACGCGTA	
	TTTGACGCGTATC	GATACGCGTCAAA	
	ATCGACGCGTCAA	TTGACGCGTCGAT	

Table 2: Top-weighted k-mers as defined by the SVR models on PBM data for selected yeast TFs. The second column shows the reverse compliments and the last column shows the PBM motifs as previously reported (Zhu et al., 2009).

TF	SVR k-mers		PSSM
Zfp740	CCCCCCCCCCCTCA	TGAGGGGGGGGGGG	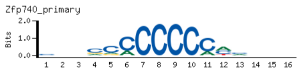
	GCCCCCCCCCCCTC	GAGGGGGGGGGGGC	
	CCCCCCCCCCCGTT	AACGGGGGGGGGGG	
	TCCCCCCCCCCCGT	ACGGGGGGGGGGGA	
	CACCCCCCCCCCAG	CTGGGGGGGGGGTG	
Bhlhb2	GGTCACGTGCCAC	GTGGCACGTGACC	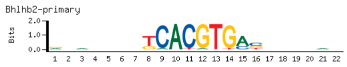
	CGGCACGTGCCTT	AAGGCACGTGCCG	
	GTCGGCACGTGAT	ATCACGTGCCGAC	
	AGGTCACGTGCCA	TGGCACGTGACCT	
	CGTCACGTGACCA	TGGTCACGTGACG	
Sp4	GTCCGCCCCCCCC	GGGGGGGGCGGAC	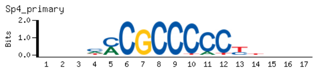
	ACCCCGCCCCCTT	AAGGGGGCGGGGT	
	TCCGCCCCCCCCG	CGGGGGGGGCGGA	
	CGCCCCCGCCCCT	AGGGGCGGGGGCG	
	GCCACGCCCCCTA	TAGGGGGCGTGGC	
Plagl1	GAGGGGGCCCCCA	TGGGGGCCCCCTC	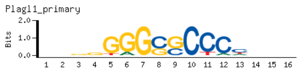
	AGAGGGGGCCCCC	GGGGGCCCCCTCT	
	AGGGGGCCCCCAG	CTGGGGGCCCCCT	
	AGGGGCCCCCTCC	GGAGGGGGCCCCT	
	CGGGGCCCCCCGA	TCGGGGGGCCCCG	
Gm397	GGTGTGTGCACAT	ATGTGCACACACC	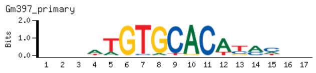
	GTGTGTGCACATT	AATGTGCACACAC	
	TGTGTGCACATTT	AAATGTGCACACA	
	CATGTGCACATAC	GTATGTGCACATG	
	ATGTGCACATACG	CGTATGTGCACAT	

Table 3: Top-weighted k-mers as defined by the SVM models on select PBM platforms (as indicated by v1 or v2) for some mouse TFs. The second column shows the reverse compliments and the last column shows the PBM motifs as previously reported (Badis et al., 2009).

References

Badis, G., Berger, M. F., Philippakis, A. A., Talukder, S., Gehrke, A. R., Jaeger, S. A., Chan, E. T., Metzler, G., Vedenko, A., Chen, X., *et al.*, 2009. Diversity and complexity in DNA recognition by transcription factors. *Science*, **324**(5935):1720–1723.

Bolotin, E., Liao, H., Ta, T. C., Yang, C., Hwang-Verslues, W., Evans, J. R., Jiang, T., and Sladek, F., 2009. Integrated approach for the identification of human HNF4α target genes using protein binding microarrays. *Hepatology*, . Advanced access online.

Georgiev, S., Boyle, A. P., Jayasurya, K., Ding, X., Mukherjee, S., and Ohler, U., 2010. Evidence-ranked motif identification. *Genome Biol*, **11**(2):R19.

Gordân, R., Hartemink, A. J., and Bulyk, M. L., 2009. Distinguishing direct versus indirect transcription factor-DNA interactions. *Genome Res*, **19**(11):2090–2100.

Harbison, C. T., Gordon, D. B., Lee, T. I., Rinaldi, N. J., Macisaac, K. D., Danford, T. W., Hannett, N. M., Tagne, J.-B., Reynolds, D. B., Yoo, J., *et al.*, 2004. Transcriptional regulatory code of a eukaryotic genome. *Nature*, **431**(7004):99–104.

Kharchenko, P., Tolstorukov, M., and Park, P., 2008. Design and analysis of chip-seq experiments for dna-binding proteins. *Nature Biotechnol*, **advanced online publication**:1351–1359. 10.1038/nbt.1508.

Siepel, A., Bejerano, G., Pedersen, J. S., Hinrichs, A. S., Hou, M., Rosenbloom, K., Clawson, H., Spieth, J., Hillier, L. W., Richards, S., *et al.*, 2005. Evolutionarily conserved elements in vertebrate, insect, worm, and yeast genomes. *Genome Res*, **15**(8):1034–1050.

Zhu, C., Byers, K., McCord, R., Shi, Z., Berger, M., Newburger, D., Saulrieta, K., Smith, Z., Shah, M., Radhakrishnan, M., *et al.*, 2009. High-resolution DNA binding specificity analysis of yeast transcription factors. *Genome Res*, **19**:556–566.

Figures

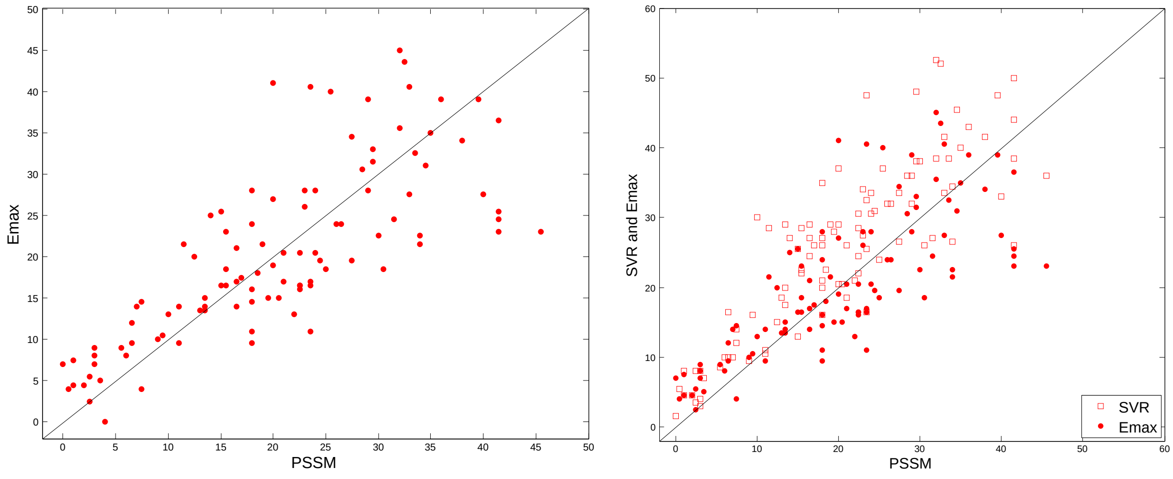

(a) (b)

Figure 1: **Detection of the top 100 PBM probes for mouse TFs.** Scatter plots show detection of the top 100 probes, ranked by intensity, within the top 100 predictions over mouse TFs (Badis et al., 2009), in cross-validation experiments over probe designs. We show PSSMs (x-axis) versus (a) maximum E-scores (y-axis) and (b) both maximum E-scores and the SVR model (y-axis).

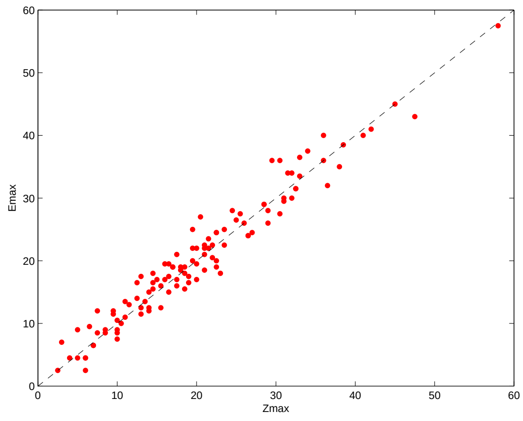

Figure 2: **E-max versus Z-max predictions.** The scatter plot shows the detection of the top 100 PBM probes using Emax and Zmax predictions for 114 mouse TF *in vitro* binding preferences.

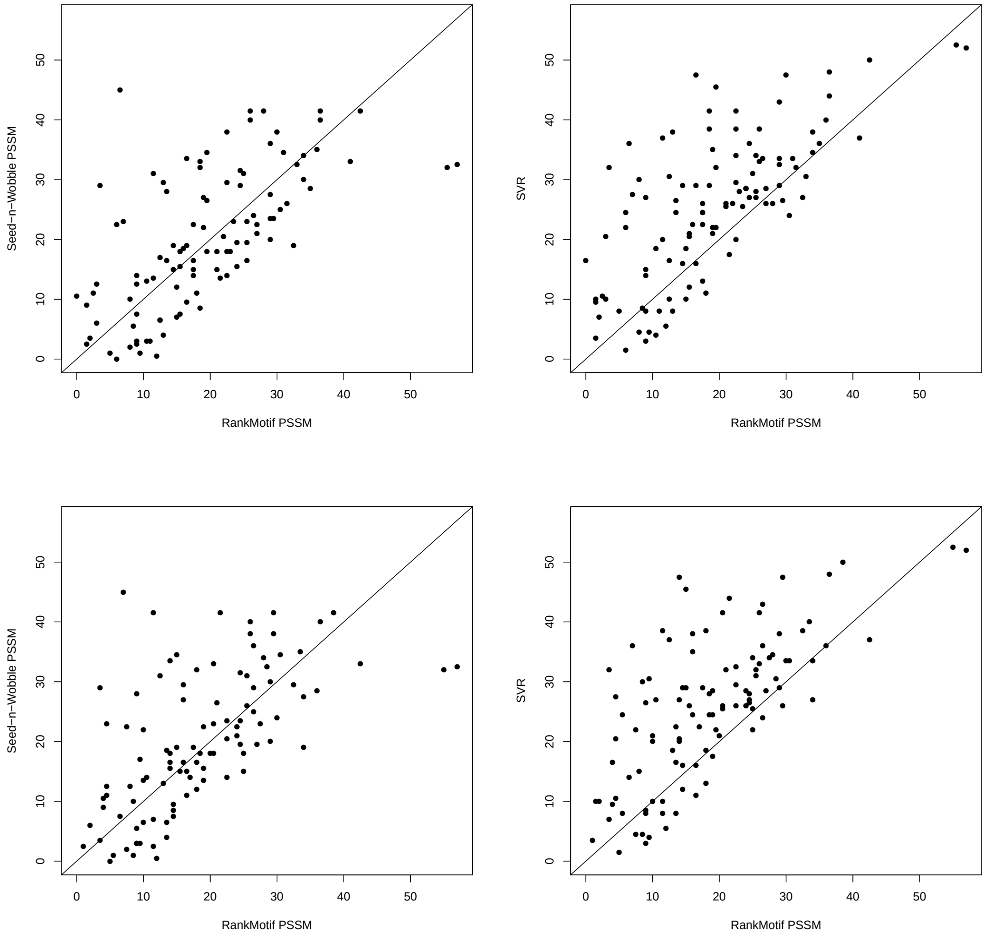

Figure 3: **Comparison with RankMotif.** The scatter plots compare the performance of RankMotif when learning a model from one PBM array design and applying it to another array design. Each plot shows the detection of the top 100 probes (averaged over the two array designs) within the top 100 predictions for each method for a data set of mouse TFs. Statistics are given (A) The best RankMotif length versus Seed-n-Wobble primary motif (SNW 47 wins, RM 55 wins; no significant difference at $p < 0.05$ threshold, signed rank test). (B) The best RankMotif length versus SVR (SVR 81 wins, RM 26 wins; $p < 3.7e-05$, signed rank test). (C) Length 10 RankMotif versus Seed-n-Wobble primary motif (SNW 54 wins, RM 48 wins; no significant difference at $p < 0.05$ threshold, signed rank test). (D) Length 10 RankMotif versus SVR (SVR 82 wins, RM 24 wins; $p < 3.4e-05$, signed rank test).

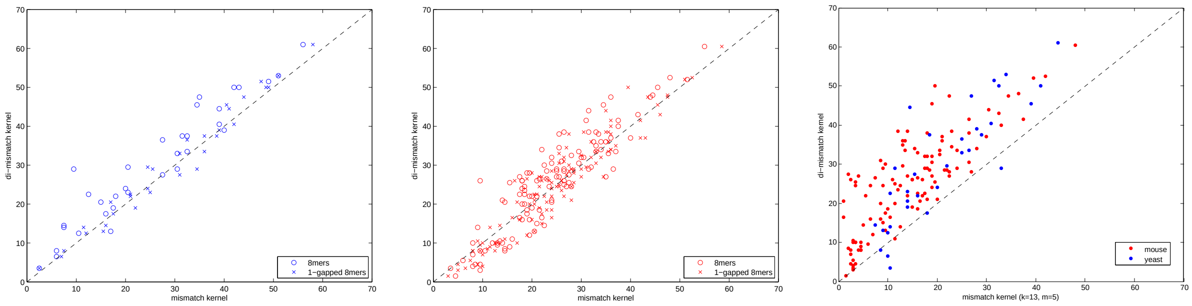

Figure 4: **Di-mismatch versus mismatch kernel.** The left plot shows the detection of the top 100 probes for 33 yeast TFs, and the middle plot shows similar results for 115 mouse TFs. The plot on the right shows results for the same yeast and mouse TFs using the mismatch kernel with $k = 13$ and $m = 5$.

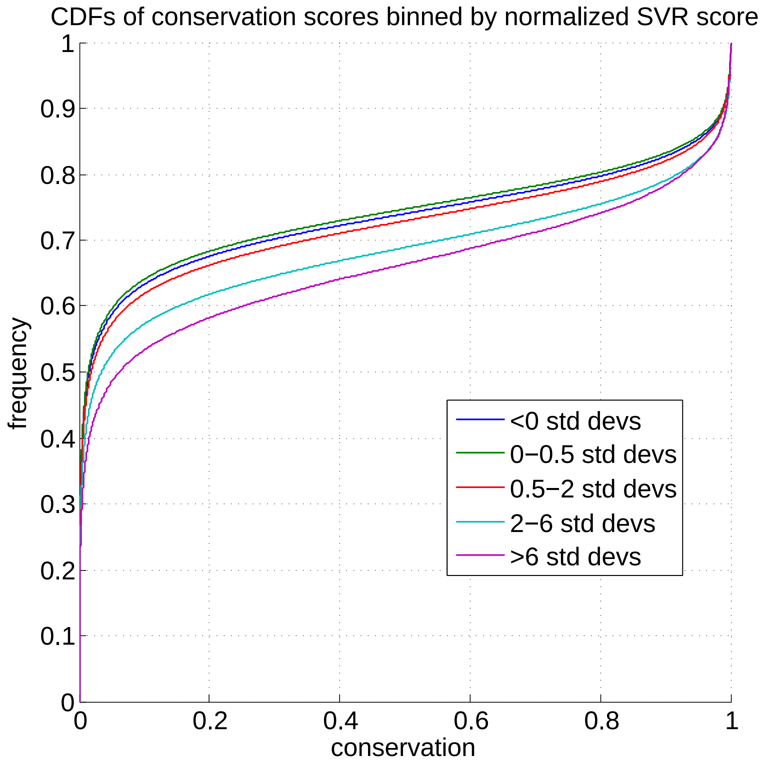

Figure 5: **Conservation levels of sequence regions near SVR peaks for 68 yeast TFs**. SVR peaks were Z-normalized and partitioned into bins of 0-0.5, 0.5-2, 2-6 and >6 standard deviations, corresponding to predictions of background, weak binding, moderate binding, and strong binding regions.

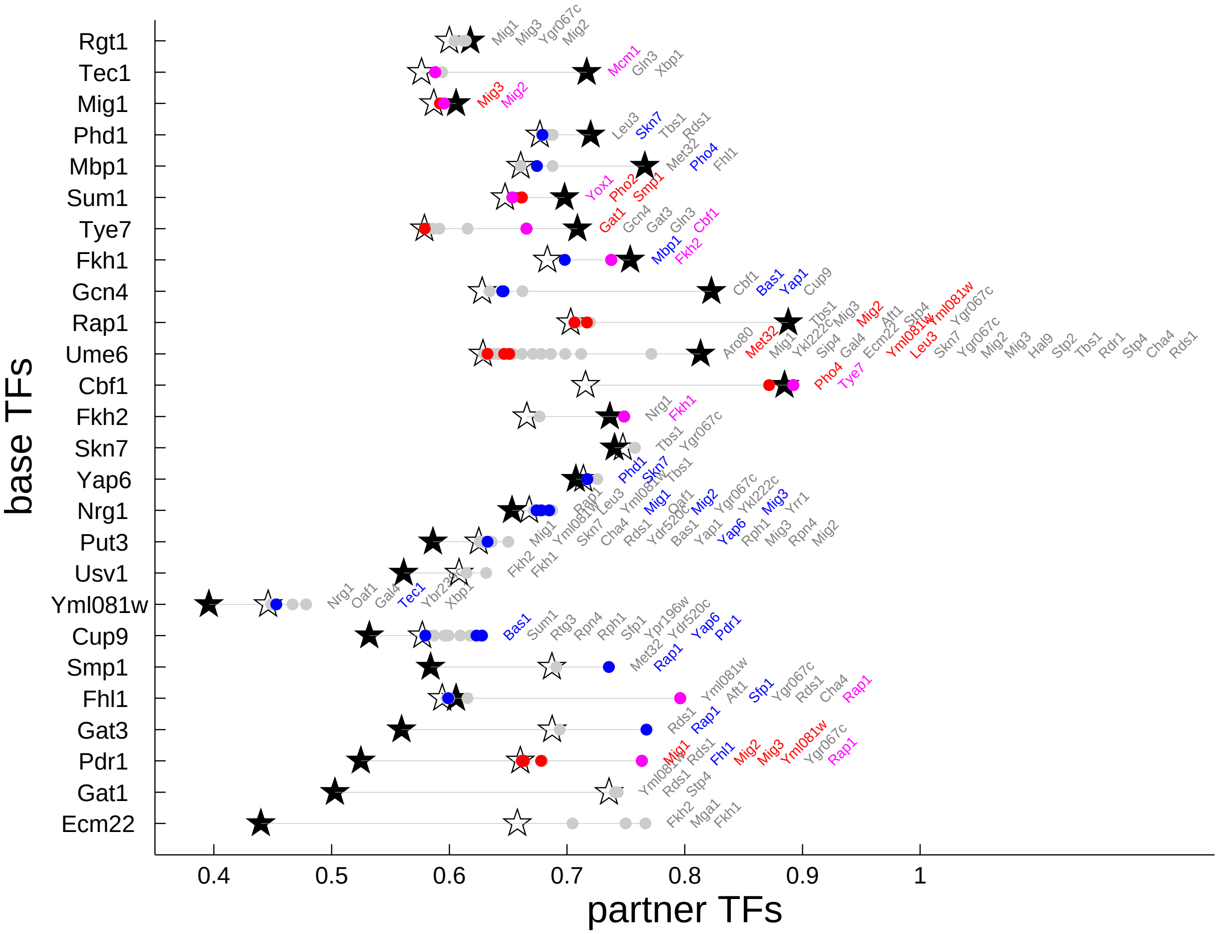

Figure 6: **Candidate interacting yeast TF pairs from ChIP-chip analysis.** Candidate binding partners are identified from an ROC analysis on the SVR models of TFs P partnering with base TFs B (y-axis). We denote the AUC obtained by applying P's SVR model to B's *in vivo* binding preferences as $f_P(B)$, and this is shown on the x-axis. A pair of TFs (B, P) was considered to be interactive if $f_P(B) > \max_i(f_{P_i}(B_{rand}))$ (indicated by empty stars), where $\max_i(f_{P_i}(B_{rand}))$ is the optimal performance obtained over all yeast TFs P_i on randomly perturbed IGRs, and validated on B's *in vivo* binding preferences. For comparison, we indicate the performance of B's SVR model on the true IGR sequences, $f_B(B)$, using solid black stars. Note that there are several instances where the black star falls before the empty star, indicating that the predicted binding preferences for B using the SVR model derived from B's PBM data, f_B, are worse than random. The performance on B of SVR models for various partners P that exceed random performance are indicated by colored dots; note that these always exceed the empty star. If partner P shares a significant overlap of its *true* bound IGRs with base B, then P is labeled in blue, suggesting that B and P bind cooperatively. If partner P shares a significant overlap of its *predicted* IGRs with base B, then P is labeled in red. In this case, it is likely that P and B prefer similar sequences and may compete for binding sites. If both conditions apply to P, then it is labeled in magenta; if neither condition applies, P is labeled in grey.

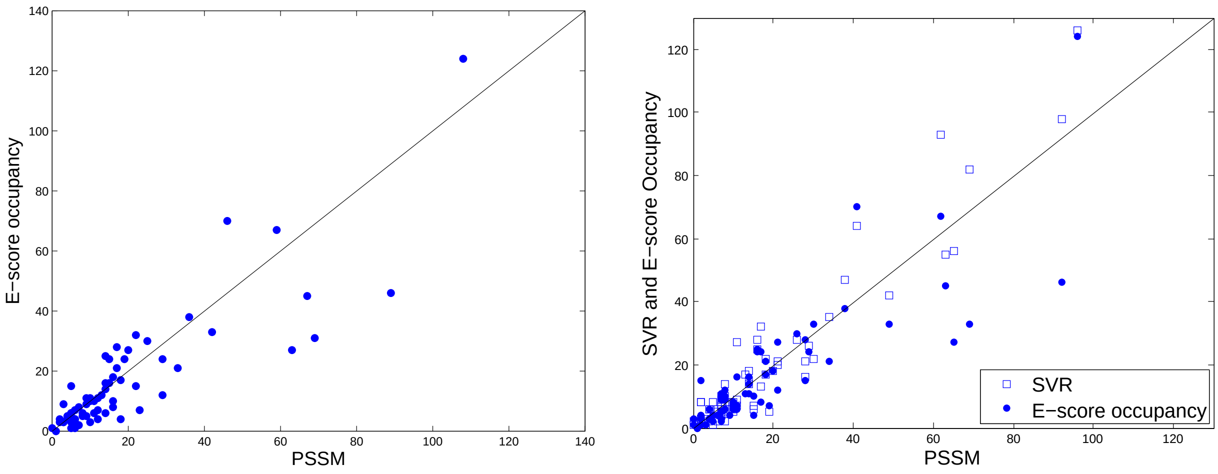

(a) (b)

Figure 7: **Detection of the top 200 IGRs for 68 yeast TFs.** Scatter plots show detection of the top 200 ranked yeast IGRs, based on ChIP chip experiments from Harbison et al. (2004), within the top 200 predictions using PSSMs (x-axis) versus (a) E-score occupancy (y-axis) and (b) both E-score occupancy and the SVR model (y-axis).

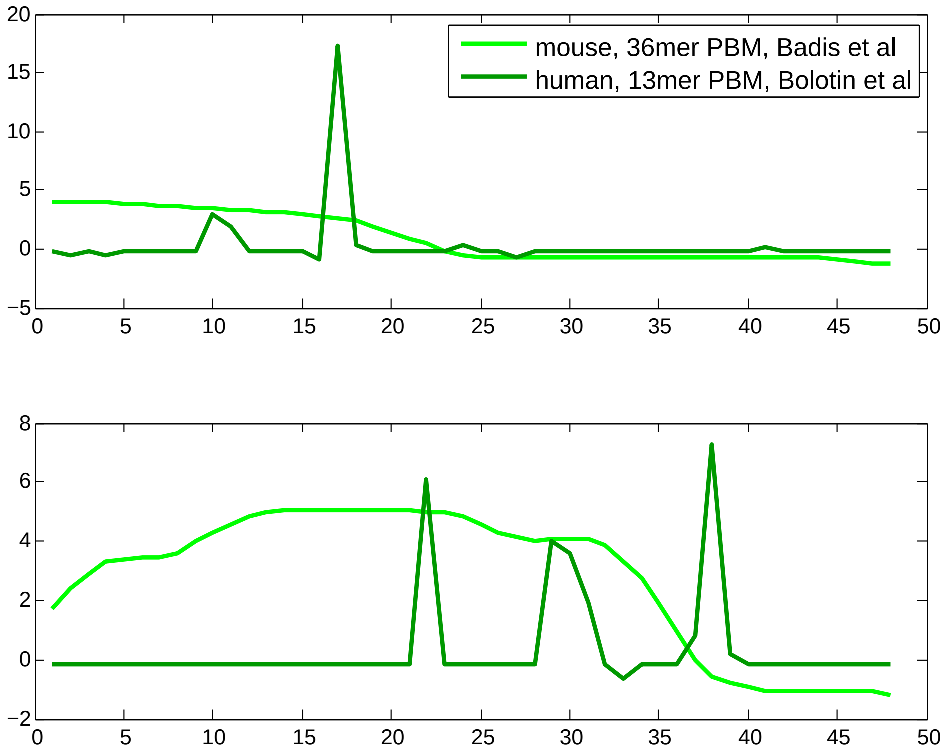

Figure 8: **SVR profiles for standard and custom PBM arrays for Hnf4a near two ChIP-seq peaks.** The top profile corresponds to a ChIP-seq peak ranked first by an SVR trained on the custom PBM array and 30th by an SVR trained on a standard PBM array. The bottom profile corresponds to a region ranked first by the standard SVR model and 458th by the custom SVR model.

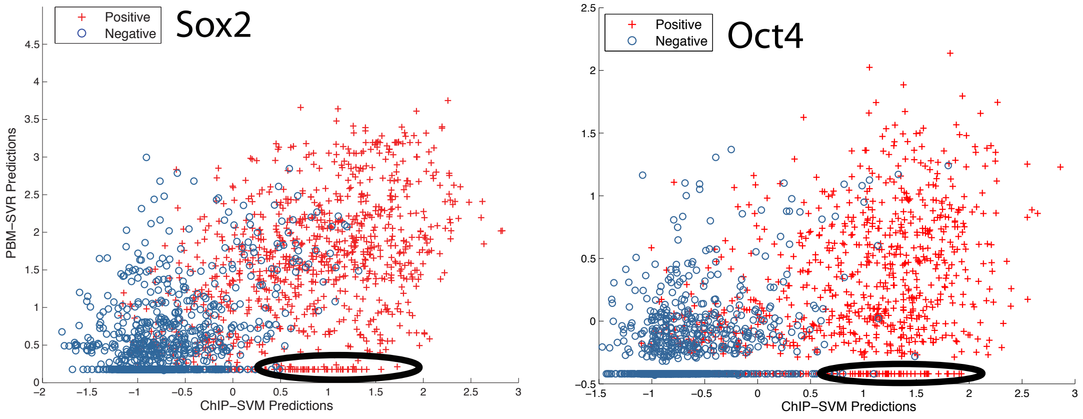

(a) (b)

Figure 9: **Scatter plots showing *in vitro* vs. *in vivo* predicted scores for Sox2 and Oct4.** The SVM trained on ChIP-seq data (x-axis) is contrasted to the PBM array (y-axis) for (a) Sox2 and (b) Oct4. The circled regions show ChIP-peaks that are correctly predicted by ChIP-derived SVM and incorrectly by PBM-derived SVR.

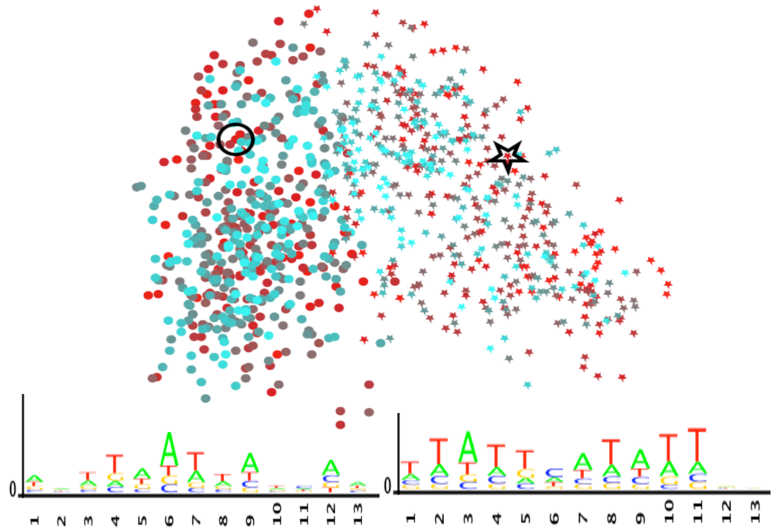

Figure 10: **Feature analysis for Pou2f1.** Visualization of k-mers contributing to the Pou2f1, similar to analysis in main paper.

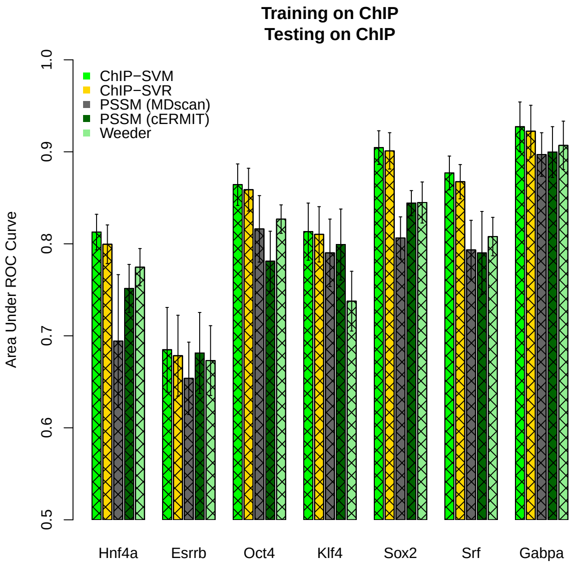

Figure 11: **Expanded analysis of ChIP-seq trained sequence models.** An expanded version of Figure 4 from the main text, with two additional bars: cERMIT PSSMs and SVR. The black lines going through the bars indicate 10-fold cross-validation results.
